# Interferon Gamma Receptor: The Beginning of the Journey

**DOI:** 10.3389/fimmu.2013.00267

**Published:** 2013-09-03

**Authors:** Cédric M. Blouin, Christophe Lamaze

**Affiliations:** ^1^Laboratoire Trafic, Signalisation et Ciblage Intracellulaires, Institut Curie – Centre de Recherche, Paris, France; ^2^CNRS UMR144, Paris, France

**Keywords:** interferon gamma receptor, interferon alpha receptor, endocytosis, endosome, clathrin, raft, JAK, STAT

## Abstract

Our view of endocytosis and membrane trafficking of transmembrane receptors has dramatically changed over the last 20 years. Several new endocytic routes have been discovered and mechanistically characterized in mammalian cells. Long considered as a passive means to terminate signaling through down-regulation of the number of activated receptors at the plasma membrane, it is now established that receptor endocytosis and endosomal sorting can be directly linked to the regulation of intracellular signaling pathways. The functional links between membrane trafficking of interferon receptors and JAK/STAT signaling have recently begun to be unraveled. These studies raise the exciting possibility that a certain level of signal specificity can be achieved through endocytosis and selective localization of the activated complexes within cellular membranes. The ongoing development of high-resolution cell imaging techniques with better spatial and temporal resolution gives new means of deciphering the inherent complexity of membrane trafficking and signaling. This should help to better comprehend the molecular mechanisms by which endocytosis and endosomal sorting of interferon receptors can orchestrate signaling selectivity within the JAK/STAT pathway that can be activated by as many as 60 different cytokines, growth factors, and hormones.

## Endocytic Processes in Mammalian Cells

Endocytosis is a highly dynamic and tightly regulated process by which solutes and macromolecules enter the cell and are distributed within intracellular compartments. This process is hijacked by several pathogens and its physiological importance well illustrated by the severity of diseases involving mutations in associated genes (1). Our view of endocytosis, and more broadly of membrane trafficking, has dramatically changed over the past 20 years. The availability of more selective tools and higher resolution cell imaging microscopy has led to the discovery and characterization of novel regulators and to the development of new concepts. Long seen as a passive means to control the number of signaling receptors at the plasma membrane, it is now clear that endocytosis and endosomal sorting can play a more direct control on the signaling outputs of several transmembrane receptors involved in fundamental cellular processes. The past decade has confirmed that in addition to the main clathrin-dependent pathway, several other endocytic pathways can operate at the cell surface of mammalian cells. The role of these alternate endocytic routes is just now beginning to emerge. We must now seek to identify the cargos that are internalized through these various pathways, to define the associated molecular machineries, and to understand the specific cellular functions that they regulate. Future work will have to integrate the molecular knowledge of endocytic sorting to other fields of research and to switch from purely descriptive to more functional understanding. In this respect, cytokine receptors, especially interferon receptors, have suffered from a relative disinterest from cell biologists. In this review, we describe the recent progress on endocytosis and endosomal sorting of signaling receptors and how this knowledge can be used as a paradigm to better understand the biological activity of interferons (IFN).

## The Classical Clathrin and Dynamin Dependent Endocytosis

Historically, clathrin-dependent endocytosis has been and still is by far the most widely studied, and thus the best understood endocytic pathway in mammalian cells. In fact, the vast majority of transmembrane receptors are endocytosed through clathrin-coated pits (CCP) (2). Clathrin-dependent endocytosis was initially described on the basis of electron microscopy studies that identified the first coated invaginated structures in the 1960s (3, 4). The minimal machinery that is theoretically required to assemble a functional endocytic structure is the structural unit clathrin, the AP-2 complex that recognizes specific motifs on the tail of endocytosed receptors, and the GTPase dynamin, which mechanically mediates the closure and the detachment of the clathrin-coated vesicle from the plasma membrane (5, 6). Nevertheless, many accessory proteins have since been shown to interact with these three historical actors so as to integrate endocytosis with other cellular machineries including the actin cytoskeleton, lipids, and signaling molecules (7–9). Two endocytic behaviors are schematically described for the initial steps of receptor uptake by clathrin-dependent endocytosis. Receptors undergoing constitutive endocytosis are internalized whether or not they have bound their ligand. This is typical of receptors that bring nutrients into the cell and best exemplified by the LDL and transferrin receptors. In contrast, receptors endocytosed through ligand-induced endocytosis undergo internalization only after binding to their cognate ligand. This is the case of most receptor tyrosine kinases (RTK) such as the EGF-R, and of G-protein coupled receptors (GPCR) that undergo endocytosis upon binding to their agonist (10, 11). It is likely that this distinct behavior relies on ligand-induced conformational change of the receptor that facilitates the interaction of otherwise hidden endocytic motifs with the AP-2 complex in the case of RTKs or β-arrestins complex in the case of GPCRs. Several aspects of the IFN-α receptor complex (IFNAR) endocytosis support this hypothesis. The resting IFNAR complex is in a conformation such that the receptor-associated Tyk2 kinase masks the classical Yxxϕ tyrosine-based endocytic motif (YVFF) in position 466 of the IFNAR1 subunit, thereby preventing its recognition by the AP-2 complex. IFN-α binding results in IFNAR1 ubiquitination, which in turn stimulates IFNAR1 internalization by exposing its endocytic motif for AP-2 binding (12). Although the endocytosis of the IFN-γ receptor complex (IFNGR) can also be stimulated through ubiquitination by the Kaposi’s sarcoma-associated herpes virus (KHSV) ubiquitin ligases K4 and K5 (13), IFNGR endogenous ubiquitination induced by IFN-γ has not been reported. IFNGR1 and IFNGR2, the two subunits of the IFNGR complex, possess putative AP-2 binding motifs. A leucine-isoleucine (LI) doublet and a typical YVSL tyrosine-based motif are present in position 270–271 and 287–290 of IFNGR1, respectively. Likewise, an YRGL motif is present on position 273–276 and a LI doublet is found on position 255–256 of IFNGR2 (14). The deletion of these motifs impairs the internalization of IFN-γ and the uptake of IFNGR2 and IFNGR1 subunits (15–18). The deletion of the corresponding LI motif on IFNGR2 does not result in a strong inhibition of its endocytosis, implying that the tyrosine-based endocytic motifs are also required for efficient uptake (15). Accordingly, it was shown in 2006 that RNAi-mediated silencing of clathrin and dynamin led to the accumulation of IFNGR1 at the plasma membrane and inhibition of IFN-γ endocytosis in several cell types (19). Whether other endocytic pathways can also contribute to the uptake of the IFNGR complex remains to be established (see below). It was recently shown that efficient IFNGR1 uptake does not depend on the LI motif but on a new 287-YVSLI-291 motif including the already identified YVSL motif and the two adjacent LI amino acids (20).

## Clathrin-Independent Endocytosis

**Figure 1:**
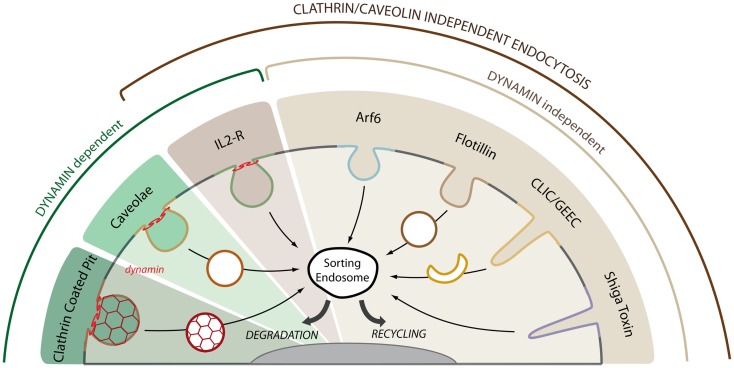
**Multiple endocytic pathways operate in mammalian cells**. Cargo proteins can enter the cell by clathrin and clathrin-independent endocytic pathways. The GTPase dynamin is required for the detachment of endocytic carriers from the plasma membrane in the clathrin, caveolae, and IL2-R pathways. The IL2-R pathways is the only clathrin and caveolae independent pathway that requires dynamin for cargo uptake. Among the other clathrin- and dynamin-independent pathways, we can distinguish between Arf6- or flotillin-dependent endocytosis, GPI-AP uptake via crescent-like intermediates (CLIC/GEEC pathway) and toxin-induced invaginations (Shiga toxin). The plasma membrane is highly plastic and a given receptor may use several of these pathways for entry and signaling. After uptake, cargo molecules are trafficked to the sorting endosome where they are either targeted to the lysosome for degradation or recycled back to the plasma membrane through recycling endosomes.

It has been now confirmed that in addition to the canonical clathrin-dependent endocytosis, several distinct endocytic pathways can simultaneously operate in mammalian cells (Figure 1) (21–23). These alternate pathways, which have been defined under the generic name of clathrin-independent endocytosis, have their own characteristics, but they also share some common features such as the association with lipid microdomains, the role of the actin cytoskeleton in cargo recruitment and vesicular scission, and their distinct regulation by the Rho family of small GTPases (Table 1).

**Table 1:** **Morphological and molecular characteristics of the different clathrin-independent endocytic pathways operating in mammalian cells**.

	Endocytosis pathway	Morphology	Protein partners	Cargo proteins
Dynamin dependent	Caveolae	Vesicular	Caveolin-1, -2, -3, cavin-1, -2, -3, -4, Src, PKC, actin	Cholesterol, glycosphingolipids, AMF, lactosylceramide, CTxB, SV40, albumin
	IL2-R	Vesicular	RhoA, Rac1, PAK1, PAK2, cortactin, N-WASP, actin	IL2-Rß, γc chain, *Clostridium* toxin, AMF
Dynamin independent	CLIC-GEEC	Tubular, crescent-like	Cdc42, Arf1, GRAF1, actin	GPI-anchored proteins, fluid-phase uptake markers
	Arf6	Vesicular	Arf6, actin	CD59, MHCI, carboxypeptidase E, β-integrins, E-cadherin
	Flotillins	Vesicular	Flotillin-1,-2, actin	GPI-anchored proteins, cholera toxin B subunit
	Toxins	Tubular	Actin	Shiga toxin B subunit, cholera toxin B subunit, SV40, galectins

### Caveolar endocytosis

Caveolae were discovered 10 years prior to CCP in mammalian cells (24, 25). Caveolae are specialized membrane invaginations that are particularly abundant at the surface of endothelial cells, muscle cells, and adipocytes, but absent in lymphoid cells and neurons (26). Caveolin-1 (Cav1) is the major constituent of caveolae and its oligomerization is sufficient to assemble a complete, functional caveola. The second isoform Cav2 is less characterized, while Cav3 is only expressed in muscle cells. The down-expression of Cav1 and Cav3, but not Cav2, is sufficient to prevent the formation of caveolae at the plasma membrane. Cavins, a new family of cytosolic proteins involved in the assembly of caveolae at the plasma membrane have been recently identified. This family includes cavin-1 or polymerase I and transcript release factor (PTRF), cavin-2 or serum deprivation protein response (SDPR), cavin-3 or sdr-related gene product that binds to C-kinase (SRBC), and cavin-4 or muscle restricted coiled–coil protein (MURC) (27). Cavins interact with caveolins in a lipid dependent manner and are required to maintain their invaginated structure. The down-regulation of cavins leads to the disassembly of caveolae and to the release of free caveolins that are subsequently degraded (28). The resembling shape of caveolae with CCP initially prompted investigators to analyze their potential ability to mediate endocytosis. Indeed, caveolae are 60–80 nm diameter cup-shaped membrane invaginations that bear a characteristic striated coat visible by electron microscopy (29). The GTPase dynamin, which mediates the mechanical release of CCP from the plasma membrane, has also been found in caveolae and is required for caveolae detachment from the cell surface. Yet, little evidence exists in support of a prominent role of caveolae in endocytic uptake in mammalian cells. Initial studies investigating the dynamics of caveolins by FRAP microscopy (fluorescence after photobleaching recovery) have revealed that the turnover of caveolins is very slow at the plasma membrane at steady state (30). Nonetheless, caveolar endocytosis can be efficiently triggered by caveolin tyrosine phosphorylation and appears to be regulated by Src kinase, protein kinase C α and actin (26). Although caveolae have the ability to recruit and concentrate various signaling molecules and effectors, no caveolar specific cargo has been identified thus far (31). Yet, several cargos can be found associated with caveolae and undergo caveolar endocytosis in a non-exclusive manner. This is the case with the autocrine motility factor (AMF) and lactosylceramide that will reach the endoplasmic reticulum after caveolar endocytosis (32, 33). It has been proposed that caveolae deliver cargo to a particular endosomal compartment called the caveosome (34). However, it has been recently shown that this compartment is a likely artifact resulting from caveolin overexpression (35). Both IFNGR and IFNAR subunits display the typical endocytic codes recognized by the clathrin-dependent endocytosis machinery, some studies however found these receptors in caveolar domains. Early electron microscopy studies showed that IFN-γ and the IFNGR1 subunit were localized both in caveolae and CCPs (36). Likewise, IFNAR was found to biochemically cofractionate with caveolin in murine cells, implying a possible association with caveolae (37). These studies then suggest that IFN receptors can be endocytosed by caveolae in addition to their uptake through CCPs. This hypothesis needs however to be confirmed by ultrastructural means and quantitative dynamic experiments under selective conditions of caveolar endocytosis inhibition.

### Non-caveolar non-clathrin endocytosis

It has long been assumed that the uptake of transmembrane receptors could only occur through CCP endocytosis (38). In the early 2000s the use of selective molecular inhibitors of clathrin-dependent endocytosis enabled the identification of the interleukin-2 receptor (IL2-R) as the first transmembrane receptor to be efficiently endocytosed in cells deprived of clathrin and caveolin (39). IL2 binding led to the association of the IL2-R with detergent-resistant membranes (DRM), the fraction collected after detergent solubilization of cellular membrane and flotation on a density gradient, reflecting the sensitivity of this clathrin-independent pathway to cholesterol and sphingolipids. The IL2-R pathway requires dynamin GTPase activity, a feature that so far distinguishes this pathway from the other clathrin-independent endocytic pathways that are dynamin-independent (see below). Another characteristic of the IL2-R pathway is the selective regulation by the Rho family of small GTPases (Table 1). Other molecular regulators have since been characterized including the p21-activated kinases PAK1 and PAK2, which can remodel the actin cytoskeleton through cortactin phosphorylation and the Wiskott–Aldrich syndrome neuronal protein N-WASP (40). The IL2-R pathway is likely to be used by other cytokine receptors including IL4, IL7, IL9, and IL15 which share the β and γ chains of the IL2-R. It was recently shown that some bacterial toxins of the *Clostridium* family can hijack the IL2-R pathway for cell intoxication (41). The AMF is endocytosed by a pathway that is regulated in a similar manner except for the sensitivity to RhoA (42). It is not known whether IFNAR and IFNGR can take the IL2-R endocytic route.

### Dynamin-independent endocytic pathways

The seminal finding in 1995 that inhibiting the dynamin GTPase does not block the overall endocytic activity of the cell raised the possibility of dynamin-independent endocytic pathways (43). Indeed, several clathrin- and dynamin-independent pathways have since been identified and characterized (Figure 1). These pathways have long been defined in negative terms due to the lack of identified regulators but more recent studies have started to identify new molecular machineries (Table 1). As for caveolae, no transmembrane receptor has yet been found to be a selective cargo of these dynamin-independent pathways. However, several transmembrane receptors are likely to use these pathways in addition to their clathrin-dependent uptake as first shown for the EGF and the TGF-β receptors (44, 45). As originally suggested by Schmid et al. (43), the dynamin-independent pathways are mostly involved in the uptake of solutes, the so-called fluid-phase uptake, or bulk flow endocytosis.

### The CLIC/GEEC pathway

This pathway is specifically involved in the uptake of GPI-anchored proteins (GPI-AP), whose insertion in the external leaflet of the plasma membrane is mediated by a glycosyl phosphatidylinositol lipidic anchor. GPI-APs are organized as monomers and/or as nanometer-scale clusters that are associated with lipid microdomains enriched in cholesterol and sphingolipids (46). GPI-AP uptake does not depend on clathrin and thereby defines a novel clathrin-independent endocytic pathway named CLIC for clathrin-independent carriers. GPI-AP are delivered to a specialized endosomal compartment called GEEC for GPI-AP enriched endocytic compartment that is distinct from the conventional Rab5 positive early endosome. CLICs display a typical morphology with a ring or crescent-like tubular shape. This pathway can be also distinguished molecularly from the IL2-R pathway since it does not require the activities of dynamin or RhoA. It is however regulated by cdc42 and Arf1 (47, 48). The recent identification of GRAF1 as a BAR domain-containing protein present on the tubular structures positive for GPI-AP and associated with cdc42 activity allows now to selectively characterize this pathway (49). The CLIC/GEEC pathway contributes to an important fraction of the overall fluid-phase uptake of the cell and its efficient recycling back to the plasma membrane.

### Flotillins

Flotillins 1 and 2 present a similar topology with caveolins and as such are also associated with lipid microdomains. However, they organize domains that are distinct from caveolar domains. Flotillin down-expression partially inhibits the uptake of cholera toxin (CTx) and of GPI-AP in murine fibroblasts (50). The flotillin endocytic pathway does not require the activity of dynamin, and appears to rely on the formation of tubular invaginations that are morphologically close to the caveolar ones. A specific cargo for the flotillin pathway remains elusive and questions continue particularly on its cellular function. After stimulation by IL6, STAT3 can be found in DRM fractions also containing flotillin in hepatocarcinoma cells (51). Whether the IFNGR and IFNAR subunits can cofractionate with the STAT molecules in flotillin positive fractions is still unknown.

### Arf6

The expression of a dominant negative mutant of the ADP-ribosylation factor ARF6 present at the plasma membrane suggested the existence of a novel clathrin-independent pathway regulated by this small GTPase (52). The GPI-anchored protein CD59 and MHC class I molecules have been shown to enter the cell through this pathway (53). Surprisingly, even though CD59 is GPI-anchored, it does not reach the GEEC endosome described for the CLIC pathway. Other cargos include carboxypeptidase E, β1 integrin, and E-cadherin. In fact, it appears that Arf6 rather regulates the recycling of these cargos to the plasma membrane.

## Role of Lipid Microdomains

### Overview

It is remarkable that all of the clathrin-independent pathways that have been described so far, including internalization through caveolae, have been associated with lipid microdomains of the raft type. Lipid rafts are membrane microdomains that result from heterogenous assemblies of certain lipids in the lateral plane of biological membranes. These domains, which are typically enriched in glycosphingolipids and cholesterol, show a high degree of lateral diffusion within the plasma membrane allowing thereby the inclusion or the exclusion of associated proteins or lipids in a highly dynamic manner. Since the raft concept postulate 15 years ago (54), many studies have attempted to better analyze the organization of these microdomains on biological membranes and to understand their cellular function. The elucidation of the plasma membrane nanoscale organization has become an intense area of investigation and to this day remains a work in progress in the field of cell biology. These studies have been comprehensively reviewed elsewhere (55, 56). Schematically, lipid rafts could serve as signaling platforms and/or endocytic devices. Most of the initial studies that have associated lipid rafts with signaling or endocytosis were based on cholesterol-binding drugs, such as methyl-β-cyclodextrin, that alter the structural composition of lipid rafts. Likewise, the association of receptors or signaling molecules with lipid rafts was assumed from their partition into DRMs. At that time, it was believed that DRMs reflected more or less faithfully the biochemical composition of lipid rafts in living cells. Today, the significance of DRM association must be revisited since more sophisticated techniques have since been available to probe the nanoscale organization of the plasma membrane with better temporal and spatial resolution.

### Lipid microdomains and signaling

**Figure 2:**
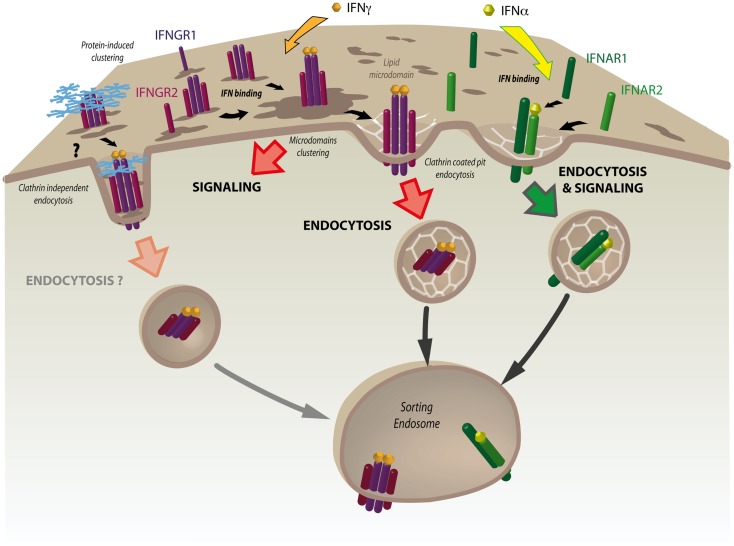
**The nanoscale organization of the IFNGR complex plays a key role in JAK/STAT signaling**. At steady state, interferon γ receptor subunits 1 and 2 (IFNGR1 and IFNGR2) are partially associated with lipid microdomains at the plasma membrane. IFN-γ binding results in rapid and dramatic increased association of the IFNGR heterotetrameric complex with these domains. IFN-γ-induced clustering is required for the initiation of JAK/STAT signaling. This is followed by the internalization of IFNGR1 and IFNGR2 through clathrin-coated pits (CCPs) and their delivery to the sorting endosome. Tetraspanins and galectins are good candidates for modulating IFNGR clustering and triggering clathrin-independent endocytosis of the IFN-γ bound receptor complex. Whether clathrin-independent endocytosis is associated with the control of IFN-γ signaling at the sorting endosome remains to be tested. In contrast to IFNGR, interferon α receptor subunits 1 and 2 (IFNAR1 and IFNAR2) form a dimeric complex that is rapidly endocytosed via CCPs after IFN-α binding. JAK/STAT signaling will occur only after the IFNAR complex has been internalized.

The intrinsic ability of lipid microdomains to assemble/disassemble in a rapid and dynamic manner is quite adapted to the control of the activation/inactivation cycles of signaling molecules at the plasma membrane, as evidenced in multiple cases (57). Two initial studies have reported that IFN-γ led to a rapid and important redistribution of the activated IFNGR complex into DRMs at the plasma membrane of different cell types (19, 58) (Figure 2). The JAK kinases and the STAT molecules have also been found associated with DRMs in these and other studies. Accordingly, the cholesterol-binding drug filipin prevented IFNGR association with DRMs and the initiation of JAK/STAT signaling by IFN-γ (19). These findings suggest that IFN-γ binding can actively control the nanoscale organization of IFNGR complexes and associated molecules of the JAK/STAT signaling pathway at the plasma membrane. In mouse cells, the IFNAR1 subunit was also detected in detergent-free isolated microdomains together with JAK and STAT (37). In human cells however, the IFNAR complex was not DRM associated and IFNAR signaling required IFNAR endocytosis through CCPs (19) (Figure 2). Whether the preferential association of murine IFNAR with DRMs is due to the absence of the tyrosine-based motif found in human IFNAR1 or whether this is caused by variations in DRM isolation protocols is unknown. A recent study confirmed the key role of IFNGR cholesterol-dependent clustering in IFN-γ biological activity (59). In the macrophages of Kala-azar patients infected by the *Leishmania donovani*, the intracellular life-cycle of the parasite leads to cholesterol quenching from the plasma membrane. As a result, IFN-γ failed to induce IFNGR localization into lipid microdomains, thus allowing the persistence of the parasite in the macrophage by lack of IFN-γ signaling. This study also identified the presence of a cholesterol-binding motif [(L/V)-X1–5-Y-X1–5-(R/K)] within the transmembrane domain (TMD) of the IFNGR1 subunit. Recently, another motif was identified in the TMD of the human and mouse IFNGR1 subunits that mediates the direct and specific interaction with sphingolipids only after IFN-γ binding (60). Whether these motifs are involved in the association of the IFNGR complex with DRMs and JAK/STAT signaling induced by IFN-γ is unknown.

This data confirms the importance of lipid-based clustering of the activated IFNGR in IFN-γ signaling both *in vitro* and *in vivo*. The challenge now is to decipher the molecular interplay occurring between lipids, the IFNGR, and the JAK/STAT signaling molecules during IFN-γ-induced IFNGR reorganization at the plasma membrane.

### Monitoring receptor nanoscale organization at the plasma membrane

Recent years have seen the emergence of new cell imaging microscopy techniques which allow the tracking of receptors dynamics at the plasma membrane with improved temporal and spatial resolution. Single cell imaging techniques such as Förster resonance energy transfer (FRET), fluorescence lifetime imaging (FLIM), and fluorescence correlation spectroscopy (FCS) allow monitoring in a dynamic and quantitative manner of protein clustering and protein–protein interactions in live cells. Single molecular tracking of nanometer-sized fluorescent objects such as Quantum Dots allows recording of the dynamics of clustered receptors in confined domains over a long time. Finally, super-resolution fluorescence microscopy has been developed during the last decade greatly improving the spatial resolution by going beyond the diffraction limit found by Ernst Abbe in 1873 (61, 62). These techniques rely on the stochastic illumination of individual molecules by photoactivated localization microscopy (PALM) or stochastic optical reconstruction microscopy (STORM). Others involve a patterned illumination that spatially modulates the fluorescence behavior of the molecules within a diffraction-limited area. This is the case with stimulated emission depletion (STED) and structured illumination microscopy (SIM). Although these techniques have increased the resolution down to 20 nm they still possess intrinsic limitations such at the time of acquisition and analysis, and the need to overexpress tagged proteins. However, these limitations are currently addressed at the level of both the microscope and fluorescent probes (63, 64). The possibility to simultaneously track the EGF receptor and EGF using two-color STED imaging is just one recent illustration of these new developments. Future improvements will certainly allow the imaging of both the receptor and associated signaling events in a dynamic manner with nanometer-scale resolution in live cells. While these techniques have not yet been applied to the IFNGR, they have been used successfully to study the dynamics of the lateral clustering of multichain immune receptor complexes such as the TCR and the BCR (65). As shown for IFNGR, ligand binding is the first step that can result in receptor clustering. Controversy exists as to whether or not IFNGR1 and IFNGR2 subunits are pre-assembled before IFN-γ binding (66). Nevertheless, as shown for the EGF-R, ligand binding can still reorganize and activate already pre-formed receptor clusters (67). In addition to ligand binding, several actors including protein–protein and protein–lipid interactions are likely to contribute to membrane dynamics and lateral clustering of signaling receptors. Tetraspanins are a family of 33 four TMD related hydrophobic proteins that are able to recognize various molecules including growth factor receptors, integrins and signaling molecules. The so-called tetraspanin web can organize a highly dynamic supramolecular network of interacting proteins that controls the lateral diffusion of signaling clusters at the plasma membrane (68). So far, no study has reported the interaction of the tetraspanins with IFN receptors. Galectins are carbohydrate-binding molecules that play pleiotropic cellular functions. Since the vast majority of signaling receptors are co- and/or post-translationally conjugated with carbohydrate moieties, galectins represent another example of molecules that could organize and control receptor clusters at the plasma membrane through a galectin-glycoprotein or -glycolipid lattice (69). Interestingly, the β-galactoside binding lectin galectin 3 was able to activate the JAK/STAT signaling pathway in an IFNGR1 dependent manner in brain-resident immune cells in mice (70). Whether this was related to the induction of IFNGR clusters has not been investigated.

The actin cytoskeleton, e.g., actin and actin-binding proteins can actively induce the formation of receptor clusters and control their dynamics at the plasma membrane (71). Actin dynamics can regulate the activity of signaling receptors either by facilitating the interaction between clusters of receptors and downstream signaling effectors or by preventing this interaction by isolating receptors from one another. This process was elegantly illustrated by CD36, a scavenger receptor responsible for the uptake of oxidized LDL in macrophages. Analysis of CD36 dynamics by single-molecule tracking showed that actin and microtubules increased the collision frequency between unliganded receptors in membrane domains thereby controlling CD36 signaling and internalization (72). Several studies have shown that receptor signaling itself can remodel the actin cytoskeleton, thus exerting a feedback loop on receptor diffusion and signaling. A non-exhaustive list of actin-mediated clustering and signaling examples include the EGF-R, the T-cell and B-cell receptors, MHC class I molecules, and GPI-AP such as CD59 (71). The potential role of the actin cytoskeleton in IFNGR clustering and signaling has not been examined. Yet, an older story had shown that antibody binding to the IFNGR1 subunit induced capping and actin colocalization (73). Interestingly, the deletion of the LI domain abolished IFNGR1 capping and redistributed IFNGR1 and actin into micropatches. Whether actin was required for IFNGR1 endocytosis or signaling has not been addressed in this study. In general, the role of the actin cytoskeleton in mediating the molecular interactions between receptors and their signaling effectors needs to be better characterized. The actin cytoskeleton is likely to interact with lipids, the other major actor in plasma membrane compartmentalization. Indeed, recent studies show that the actin cytoskeleton can affect lipid microdomain formation and dynamics, whereas cholesterol can modulate actin nucleation and dynamics (57).

### Lipid microdomains and endocytosis

Besides their role in signaling, recent studies have unveiled a new function of lipid microdomains in endocytic trafficking. One puzzling questions that has long remained unresolved in clathrin-independent endocytosis is to understand how the recruitment of cargo into endocytic carriers and the tubulation of the plasma membrane could occur in the absence of the AP-2/clathrin coat and dynamin, respectively (22). This novel aspect of lipid microdomain function has been revealed by pioneering studies on the endocytosis of Shiga toxin (STx), a bacterial toxin produced by *Shigella dysenteriae* which enters the cell by clathrin-independent means after binding to its specific receptor, the glycosphingolipid Gb3. In order to minimize the energy resulting from local perturbations on the plasma membrane, lipid domains will tend to fuse together, thereby bringing their cargo into larger domains (74). Thus, Gb3 binding of the B subunit of STx, which has a characteristic pentameric structure, leads to the compaction of the outer leaflet of the plasma membrane. It results in local asymmetries which are translated into an important inward negative curvature of the plasma membrane inside the cell (75). The concentration of cargo into those domains can be actively induced by cortical actin as shown for the GPI-AP monomers and clusters (76). Cholesterol plays a stabilizing role for the GPI-AP homodimers that would otherwise only assemble transiently in its absence (77). The invagination of lipid microdomains allows the reduction of the energy at the boundary interface through the line tension process (78). Line tension is a fundamental player in the scission of vesicles in the absence of dynamin. In this case, actin polymerization can reorganize the membrane by assembling distinct lipid domains whose boundary is energetically more favorable to membrane scission (79). In addition to their endocytosis through active reorganization of lipid domains, CTx and STx B can also enter the cell through caveolae and CCPs, respectively. Although most IFNGR are internalized by CCPs (19), it is still possible that according to the cell type or IFN-γ concentration, IFNGR could be endocytosed through a similar process involving the active clustering of IFNGR through the actin cytoskeleton or by some unidentified selective cross-linker molecules. As discussed above, tetraspanins or galectins are good candidates (Figure 2).

## Endocytosis and Signaling

In the context of intracellular signaling, endocytosis allows the rapid and efficient decrease in the number of activated receptors at the plasma membrane. In addition to this classical role, pioneering studies on the EGF-R have established almost 20 years ago that receptor endocytosis could also actively control the signaling pathways activated by EGF in a more direct manner (80). Following studies have established the key concept of the “signaling endosome,” which reflects the finding that endosomes are not simply passive recipients where internalized receptors can accumulate but instead serve as sorting stations where signaling initiated at the plasma membrane can be amplified or terminated (81). Numerous studies have since illustrated the importance of membrane trafficking in the control of intracellular signaling through temporal and spatial compartmentalization of signaling receptors and downstream effectors (65). This particular aspect of membrane trafficking has been overlooked for the IFN-Rs and the classical view of signaling, where effectors interact in a linear manner from the plasma membrane to the nucleus, has long prevailed. Accordingly, inhibition of clathrin-dependent machinery had no effect on the initiation of JAK/STAT signaling and the antiviral and antiproliferative activities induced by IFN-γ (19). Instead, as discussed above, JAK/STAT signaling relies on IFN-γ-induced IFNGR clustering at the plasma membrane. Thus, it is likely that STAT1 is first recruited to IFNGR positive lipid microdomains to be phosphorylated at the plasma membrane, then released to the cytoplasm *en route* to the nucleus prior to the uptake of the IFNGR complex by clathrin-dependent endocytosis. This is in contrast to the IFNAR complex, which also enters the cell by CCPs and shares some of the JAK/STAT effectors with the IFNGR complex, but is fully dependent on clathrin-dependent endocytosis for signaling. Therefore, the nanoscale organization of the activated IFN-R at the plasma membrane allows a clear dichotomy between IFN-α and IFN-γ for JAK/STAT signaling (Figure 2). In T lymphocytes, the mutation of the IFNGR2 LI endocytic motif led to cell surface accumulation and increased STAT1 activation further demonstrating the role of IFNGR localization at the plasma membrane for the activation of JAK/STAT signaling (15).

### Signaling regulation through caveolae

Early electron microscopy studies have found IFN-γ and the IFNGR1 subunit to be localized into caveolae in human lymphoma cells (36). Whether the IFNGR present in caveolae are activated and internalized remains unknown. As mentioned above, caveolae are rather inefficient for endocytosis and it is therefore more likely that caveolae control IFN-γ-induced signaling through IFNGR confinement at the plasma membrane. Caveola structure could allow specific interactions with the IFNGR complex and/or associated signaling molecules. The N-terminal domain of Cav1 presents a so-called scaffolding domain (CSD), composed of a stretch of 20 amino acids (residues 82–101) that interacts with cholesterol at the plasma membrane and is required for the oligomerization of caveolins (26). Based on pioneering studies with eNOS, it has been hypothesized that the CSD could interact with a corresponding caveolin binding motif (CBM) that has been found in several signaling molecules. The CSD would exert a negative regulation on interacting signaling effectors. IFNAR and IFNGR subunits do not present a classical CBM motif, yet it remains possible that some signaling downstream effectors are modulated through this interaction. Interestingly, it has been suggested that Cav1 could act as a suppressor of cytokine signaling (SOCS) by inhibiting the kinase activity of some JAK family members (82). JAK1 and JAK2 are good candidates since each contains two typical CBM motifs, one on the kinase domain and another on the pseudokinase domain. Recently, a re-examination of the structure of these motifs has questioned their role in signaling (83). The recent possibility to use cell permeable inhibitory peptides of the CSD motifs should help to assess the true function of this domain in caveolae-dependent signaling (84).

### Plasticity of the plasma membrane

The plasma membrane possesses an intrinsic high level of plasticity and the IFNGR complex has been localized to distinct specialized areas of the plasma membrane including CCPs, caveolae, and lipid microdomains. Each of these locations could carry distinct kinetics of receptor uptake, distinct intracellular distributions, and thus distinct signaling outcomes. With the exception of caveolae, few studies, if any, have addressed the possible regulation of receptor signaling by the clathrin-independent pathways. Noteworthy, after inhibition of clathrin-dependent endocytosis, there still remains a residual fraction of IFN-R that can enter the cell (19). Whether this reflects clathrin-independent endocytic possibilities and/or alternate control of signaling for a minor fraction of receptors remains to be established. More sensitive techniques will probably reveal if some of the IFNGR clusters can also be endocytosed through lipid microdomains in a process similar to the uptake of Shiga and cholera toxins. It would be also interesting to analyze whether STAT1-independent signaling may depend on IFNGR clathrin-dependent endocytosis and the presence of the endocytosed IFNGR in the endosome (Figure 2). Finally, one can imagine that IFN-R may follow distinct endocytic pathways according to the cell type. As mentioned above, IFNGR was localized in caveolae in hepatocytes. This is unlikely to happen in lymphoid cells that are devoid of caveolae. Future studies are clearly needed to correlate endocytic sorting and signaling specificities within different cellular contexts.

## Concluding Remarks

Studies in membrane biology over the past decade have started to reveal the increasing complexity of plasma membrane organization at the nanoscale level. Endosomes represent an important extension of the plasma membrane for the control of receptor signaling. The inherent plasticity of the plasma membrane combined with the intrinsically high dynamics and connectivity of the endosomal network multiplies the possibilities of controlling in both time and space various aspects of receptor behavior such as clustering, internalization, and intracellular distribution. Recently, new structural data on IFNAR have shed light on how two distinct IFNs, IFNα2 and IFNω, can elicit different receptor-ligand structural interactions that control complex stability and signal initiation (85). Whether ligand discrimination may lead to distinct endocytosis and trafficking outcomes and thereby initiate distinct signaling is an exciting possibility that remains to be tested. Likewise, distinct trafficking pathways may be associated to the antiviral or antiproliferative activities of IFNs with the corresponding activation or inhibition of selective genes. The recent possibility to use super-resolution microscopy, high throughput assays, and quantitative proteomics provides investigators with new means to have a more comprehensive insight into membrane dynamics and signaling. The future challenge is to integrate our current understanding of signaling and membrane trafficking with other cellular functions such as cell development, cell mechanics, cell migration, host-cell pathogens interactions, and other disciplines such as immunology, biophysics, metabolism, and ultimately pathophysiology. Our knowledge of IFNGR trafficking is still in its infancy and many questions remain unanswered. It is clear that more work is required to understand how membrane and endosome dynamics can control the signaling outputs of the IFNGR. This is indeed just the beginning of the journey for the IFNGR complex.

## Conflict of Interest Statement

The authors declare that the research was conducted in the absence of any commercial or financial relationships that could be construed as a potential conflict of interest.
